# Integration of interactive, multi-scale network navigation approach with Cytoscape for functional genomics in the big data era

**DOI:** 10.1186/1471-2164-13-S7-S24

**Published:** 2012-12-07

**Authors:** Thanet Praneenararat, Toshihisa Takagi, Wataru Iwasaki

**Affiliations:** 1 Department of Computational Biology, the University of Tokyo, Kashiwa, Chiba, 277-8568, Japan; 2 National Bioscience Database Center, Japan Science and Technology Agency, Chiyoda, Tokyo, 102-0081, Japan; 3 Center for Information Biology, National Institute of Genetics, Mishima, Shizuoka, 411-8540, Japan; 4 Current address: Atmosphere and Ocean Research Institute, the University of Tokyo, Kashiwa, Chiba, 277-8564, Japan

## Abstract

**Background:**

The overwhelming amount of network data in functional genomics is making its visualization cluttered with jumbling nodes and edges. Such cluttered network visualization, which is known as "hair-balls", is significantly hindering data interpretation and analysis of researchers. Effective navigation approaches that can always abstract network data properly and present them insightfully are hence required, to help researchers interpret the data and acquire knowledge efficiently. Cytoscape is a *de facto* standard platform for network visualization and analysis, which has many users around the world. Apart from its core sophisticated features, it easily allows for extension of the functionalities by loading extra plug-ins.

**Results:**

We developed NaviClusterCS, which enables researchers to interactively navigate large biological networks of ~100,000 nodes in a "Google Maps-like" manner in the Cytoscape environment. NaviClusterCS rapidly and automatically identifies biologically meaningful clusters in large networks, e.g., proteins sharing similar biological functions in protein-protein interaction networks. Then, it displays not all nodes but only preferable numbers of those clusters at any magnification to avoid creating the cluttered network visualization, while its *zooming *and *re-centering *functions still enable researchers to interactively analyze the networks in detail. Its application to a real *Arabidopsis *co-expression network dataset illustrated a practical use of the tool for suggesting knowledge that is hidden in large biological networks and difficult to be obtained using other visualization methods.

**Conclusions:**

NaviClusterCS provides interactive and multi-scale network navigation to a wide range of biologists in the big data era, via the *de facto *standard platform for network visualization. It can be freely downloaded at http://navicluster.cb.k.u-tokyo.ac.jp/cs/ and installed as a plug-in of Cytoscape.

## Background

The exponentially increasing amount of functional genomics data is significantly inhibiting researchers from making sense of these data [1]. Network visualization is widely used to represent such data (e.g., protein-protein interactions and gene co-expressions); however, it does not work effectively with the big data due to the jumble of tangled edges ("hair-balls"). Instead of being helpful to biologists, such network representations cannot be visually interpreted or further analyzed to extract meaningful biological facts.

To overcome this problem, effective navigation approaches that can abstract data properly and present them insightfully at any magnification are required [2]. We previously developed an interactive, multi-scale navigation method for large biological networks [3]. This method, which is similar to online mapping services such as Google Maps, can rapidly provide appropriately abstracted views at any magnification and enable researchers to effectively interpret networks.

Cytoscape is an OS-independent, *de facto *standard platform for network visualization and has many users around the world [4]. In addition to its built-in sophisticated features, users can easily extend Cytoscape by loading extra plug-ins. Herein, we describe our development of NaviClusterCS, which enables researchers to navigate biological networks in a Google Maps-like manner in the Cytoscape environment. Within large networks of ~100,000 nodes, NaviClusterCS rapidly finds biologically meaningful clusters, which are sets of nodes that are densely connected to each other and/or share similar biological functions (see Implementation). For example, in the case of protein-protein interaction networks, such clusters comprise proteins forming protein complexes or acting in related signaling pathways. NaviClusterCS automatically displays only the appropriate numbers of those clusters, resulting in an "abstracted view", instead of every internal node to always provide insightful and visually interpretable information. Moreover, users can flexibly investigate the networks by gradually zooming toward the level of individual nodes (*zooming in*), turning back to a broader or more abstracted view (*zooming out*), or seeing clusters around nodes/clusters of interest (*re-centering*).

## Implementation

NaviClusterCS was implemented as a Cytoscape plug-in in Java, and is available at http://navicluster.cb.k.u-tokyo.ac.jp/cs/. Three files are required for running NaviClusterCS, a node list file, an edge list file, and a property information file. The node list file and the edge list file together describe a network dataset, and the property information file contains extra information for the property-based clustering.

In detail, the node list file is a text file that describes node names, database names (e.g., TAIR for *Arabidopsis *genes), IDs used in those databases, and their annotated property information. The database information is used to provide URL links to the relevant pages describing node details, which appear as context-sensitive menus when users right-click on nodes. The property information is described as sets of *property terms *that represent attributes of the nodes, e.g., Gene Ontology (GO) terms [5] for genes and proteins. The edge list file consists of information about connected pairs of nodes and the weights of the connections; these weights describe how strongly the nodes are connected. The property information file describes the property terms' IDs, names, display names (used in labeling clusters in abstracted views), namespaces, default weights, and their parent terms. Terms with heavier weights are treated as more important properties. Each term belongs to one namespace, e.g., biological process in GO. Using this namespace information, researchers can put heavier weights on terms in certain namespaces at once, thus grouping nodes that have related terms in those particular namespaces. If parent terms are provided for each term, these parent terms are automatically assigned to the nodes that the "child" term annotates. In the case of GO, the *is_a* and *part_of* relationships are handled by this entry.

A sample dataset provided in NaviClusterCS is an ATTED-II dataset of 22,447 nodes and 189,546 edges [6], which is a large co-expression network of *Arabidopsis *genes. A default property information file is derived from the GO annotation file in the TAIR database [7] at 15 April 2011, thus each term belongs to one of the three namespaces (biological process, molecular function, or cellular component). For this property information file, the default weights are terms' depths in the GO hierarchy; this treats more specific terms as more important properties.

When large networks are loaded in NaviClusterCS, as described above, it rapidly locates biologically meaningful clusters in them, where clusters are sets of nodes that are densely connected and share similar properties. The underlying components of NaviClusterCS are (i) an ultrafast graph clustering component, (ii) a property-based clustering component, and (iii) a visualization component that connects to the Cytoscape canvas. Each component is described in detail below.

### Ultrafast graph clustering component

First, NaviClusterCS abstracts the whole network using ultrafast graph clustering to detect topologically densely connected regions, which may correspond to biologically meaningful clusters, such as groups of genes playing related roles. More precisely, it groups the nodes where connections of nodes *within *the sets are denser than the connections *between *nodes inside and outside of the sets. This metric is called the modularity or Q function [8,9], whose definition is $Q=\frac{1}{2m}\sum_{i,j}\left[A_{ij}-\frac{k_{i}k_{j}}{2m}\right]\delta \left(c_{i},c_{j}\right)$, where *A_ij _*is the weight of the edge between node *i *and node *j*, $m=\frac{1}{2}\sum_{ij}A_{ij},k_{i}=\sum_{j}A_{ij}$ is the sum of the weights of all edges connected to node *i, c_i _*is the community to which node *i *is assigned and *δ*(*u, v*) = 1 if *u *= *v *and 0 otherwise. We implemented an algorithm for quickly identifying clusters of high modularity in huge networks [3,9] as follows:

(1) Starting from the state that each node belongs to a cluster different from every other node, for each node the algorithm considers its neighbors' clusters and moves the node to a neighboring cluster. The cluster to be joined is determined by choosing the movement that results in the highest positive modularity gain among all possible movements to the node's neighboring clusters. If no movements result in a positive gain in modularity, the node is not moved. This process is repeated until no members are added to/removed from any clusters, ultimately yielding clusters with the maximum local modularity.

(2) Every cluster from phase 1 is then treated as a new node. For each pair of new nodes, an edge connecting them exists if there is at least one edge between any member of one of the new nodes and any member of the other. Edge weights are determined based on the number of previous edges. Self-loops are drawn on nodes to represent corresponding edges between members of the same clusters.

The output of phase 2 is then fed back to phase 1 and the algorithm iteratively runs these two phases until no additional changes are made. This component works with huge networks of about 100,000 nodes within a few seconds [3,9] and, thus, is highly suitable to be applied to large and complicated biological networks.

### Property-based clustering component

Recent investigations have revealed that, hub-like nodes tend to connect with low-degree nodes and the majority of nodes interact with only a few partners in some common biological datasets [10]. In these cases, large and densely connected regions are relatively rare, whereas small, densely connected modules are more frequently found. Therefore, abstraction by the first component can be inadequate. Property-based clustering can then be invoked to further abstract the network to a manageable level, allowing for visual interpretation [3]. This clustering automatically groups clusters with similar biological properties by using property information (e.g., GO terms often annotated to biological entities).

Let *N *be the number of nodes in the original input graph and *L *be the number of clusters created in the previous step (LCs). For each *n*, where 1 ≤ *n *≤ *N*, node *v_n _*has a set of terms, *T*(*v_n_*), that denotes the properties of the node (e.g., a set of GO terms). A weight, *w*(*t*), is given to each term *t *to quantify its importance (e.g., properties that are rare and/or of particular interest to researchers may be given higher weights). Let $T_{all}\equiv {⋃}_{1\leq n\leq N}T\left(v_{n}\right)$ and *T_all_* = {*t_j_*|1 ≤ *j* ≤|*T_all_*|}. For each LC, *LC_l_*, where 1 ≤ *l *≤ *L*, let *Prop*(*t*, LC_*l*_) = |{*v* ∈ LC_*l*_|*t* ∈ *T*(*v*)}|/|LC_*l*_|. The property vector for LC*_l _*or **PV**(LC*_l_*) is a |*T_all_*|-dimensional vector whose *j*-th element is the score of term *t_j_*, which is calculated as *w*(*t_j_*) *Prop *(*t_j_*, LC*_l_*). In our implementation, term *t_h _*is the property term for labeling LC*_l_*, where *w*(*t_h_*) *Prop*(*t_h_*, LC_*l*_) ≥ *w*(*t_j_*) *Prop*(*t_j_*, LC_*l*_), ∀ *j*, 1 ≤ *j* ≤ |*T_all_*|. Next, the similarity between two LCs, LC*_a _*and LC*_b_*, is given as a normalized dot product of the two property vectors *Sim*(**LC**_*a*_, **LC**_*b*_) = **PV**(**LC**_*a*_)·**PV**(**LC**_*b*_)/|**PV**(**LC**_*a*_)||**PV**(**LC**_*b*_)|. The LCs having similar property vectors are grouped by the Farthest First Traversal *K*-center (FFT) algorithm [11]. The FFT algorithm is a complexity-reducing variant of the *K-*means algorithm, where the initial *K *cluster centers are chosen as follows. The first center (vector) is chosen randomly and each remaining center is determined by choosing the vector farthest from the set of already chosen centers. The rest of the vectors are assigned to the cluster to which they are most similar.

The resulting clusters are used instead of those generated by the first component. The property-based clustering component offers two advantages: (i) the preferred number of nodes/clusters to be shown on the canvas can be directly controlled by the parameter *K*; and (ii) the property information used by this component carries biological meaning, so the produced clusters would be highly intuitive.

### Visualization component

The resultant clusters/nodes are displayed on the Cytoscape canvas along with the meta-edges and property edges, which represent the number of edges existing between any members of two clusters and the similarities between their properties, respectively. At this stage, users can interactively zoom, move laterally beyond cluster boundaries, and/or focus on an arbitrary set of nodes/clusters.

NaviClusterCS provides *zooming *and *re-centering *functions, which work similarly to those of online mapping services such as Google Maps. The *zooming *function takes all node members of the selected clusters as input, performs the two-stage clustering, and then displays the abstracted network. The *zooming *can be performed on more than one cluster at a time. Given user-defined nodes/clusters, the *re-centering *function executes the clustering on all nodes whose geodesic distances to the selected nodes/clusters are not greater than a provided value. This function corresponds to panning maps to see surrounding regions in web mapping services. By changing the geodesic distance, both fine and coarse visualization centered on the selected nodes/clusters can be created.

## Results and discussion

### Comparison with existing network visualization tools

Table 1 shows the comparison of NaviClusterCS with related network visualization tools in aspects of cluster generation means, multi-scale navigation support, purpose of use, development architecture, measures for handling overwhelming visualization (insufficient clustering), and support for flexible navigation beyond cluster boundaries. Apart from Cytoscape plug-ins, we also include stand-alone and web-based tools here. Most of them support automatic cluster generation using various underlying clustering or communities identification algorithms, except for VisANT [12], Cellular Overview [13], and GenePro [14]. Some provide multi-scale network navigation in each own way. For instance, in contrast to the intuitive zooming style adopted by NaviClusterCS, clusterMaker [15] support multi-scale navigation via dendrograms, whose parts, when selected by users, are reflected in the network view of Cytoscape. Whereas most tools aim at visualizing various types of biological networks, MODEVO [16], RobinViz [17], Cellular Overview [13], and GenePro [14], are tailored for specific types of networks, such as interaction networks and metabolic networks. Among all the tools shown in Table 1, only NaviClusterCS and RobinViz offer flexible navigation across created clusters. RobinViz implements the 2-hop neighborhood operation, where neighbors that are 2-hop far from selected nodes are gathered and visualized. NaviClusterCS, however, goes further by allowing for gathering and visualizing more than 2-hop neighbors, via the re-centering function. This can be done without producing cluttered visualization, as it automatically clusters the resultant networks after the re-centering function is invoked. Notably, adopting the property-based clustering, NaviClusterCS is the only tool that imposes measures for handling the case of overwhelming visualization due to insufficient clustering.

**Table 1:** A comparison table of related network visualization tools.

Tools / Features	Cluster Generation	Multi-Scale Navigation	Purpose of Use	Architecture	Flexible Navigation Beyond Cluster Boundaries	Measures for Handling Insufficient Clustering
NaviClusterCS	Automatic and Extremely Fast	Yes	Generic	Cytoscape Plug-in	Yes (via Re-centering)	Property-Based Clustering
CyOog (Power Graph) [22]	Automatic	Yes (Power Nodes)	Generic	Cytoscape Plug-in	No	No
clusterMaker [15]	Automatic	Yes (Dendrogram)	Generic	Cytoscape Plug-in	No	No
CyClus3D [23]	Automatic	No	Generic	Cytoscape Plug-in	No	No
NeMo [24]	Automatic	No	Generic	Cytoscape Plug-in	No	No
BioLayout Express^3D ^[25]	Automatic	No	Generic	Stand-Alone	No	No
jClust/Medusa [26,27]	Automatic	No	Generic	Stand-Alone	No	No
MODEVO [16]	Automatic	No	Protein Interaction Network	Cytoscape Plug-in	No	No
RobinViz [17]	Automatic	No	Protein Interaction Network	Stand-Alone	Yes (2-Hop Neighborhood)	No
VisANT [12]	Manual	Yes (Metanodes)	Generic	Stand-Alone	No	No
Cellular Overview [13]	Manual and Stored in Databases	Yes (Zooming User Interface)	Metabolic Network	Web-Based	No	No
GenePro [14]	Manual	No	Interaction Network	Cytoscape Plug-in	No	No

### User interface

**Figure 1:**
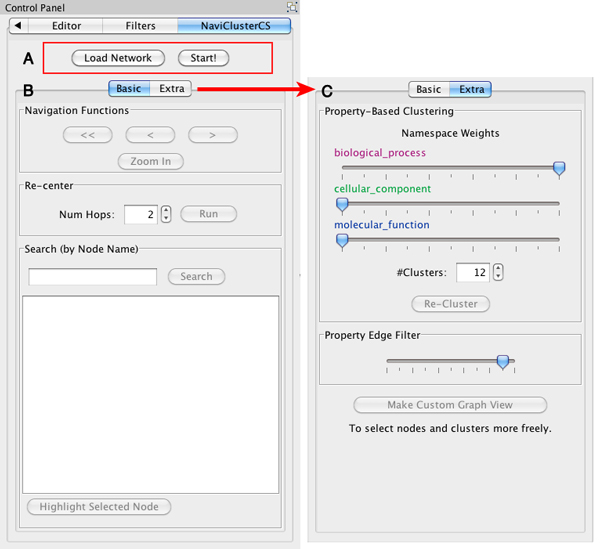
**User interface of the control panel of NaviClusterCS**. The control panel of NaviClusterCS is mainly composed of two buttons for loading network data and starting clustering the network (A), and two tabs for performing various operations on the network. The *basic tab* allows for graph view navigation, zooming, re-centering, and searching (B). The *extra tab* allows for namespace weight adjustment, network re-clustering, property edge filtering, and custom view creation (C).

The control panel of NaviClusterCS comprises two buttons, the *Load Network *button and the *Start *button, and two tabs, the *basic tab *and the *extra tab *(Figure 1). The *Load Network *button is used to load new network data and the *Start *button invokes the two-stage clustering on the network. The *basic tab *allows users to navigate views back and forth, zoom in on clusters of interest, re-center the network on clusters/nodes of interest, and search for a node of interest. In the *extra tab*, users can adjust namespace weights, re-cluster the network, refine the filter of property edges, and create new custom views. Data Panel is hidden by default to give more space for resultant networks generated by NaviClusterCS.

**Figure 2:**
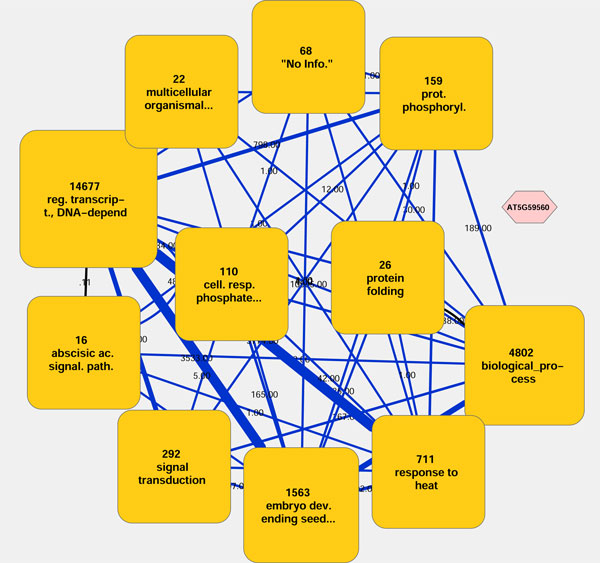
**The abstracted view of the ATTED-II dataset with 22,447 nodes and 189,546 edges**. The rounded squares are clusters and the hexagon is a gene that is not contained in any cluster. Because the gene *AT5G59560 *has no connections with any genes in this network, it appears as an unconnected node. The numbers shown above the clusters represent the number of nodes within the clusters, and the labels following the numbers are the abbreviated GO terms that get the highest score and thus best describe the properties of the clusters. Meta-edges are drawn as blue lines between any two clusters that have at least one edge between at least one member in each of the two clusters, with the numbers next to the meta-edges representing the total numbers of all edges existing between the members of the two clusters. The total numbers are also reflected by the thicknesses of the meta-edges.

Users can start with loading a node list file and an edge list file via the *Load Network *button of the *basic tab *or via the import functions of Cytoscape. Besides, users can use their networks loaded onto Cytoscape as well, provided that there exist all required attributes attached to the nodes and edges of the networks, such as an attribute named "weight" for edges. For detail, see the online manual at http://navicluster.cb.k.u-tokyo.ac.jp/cs/. After loading, the users can run the two-stage clustering (see Implementation) on the network by clicking the *Start *button (Figure 1A). A resultant abstracted view is illustrated in Figure 2. When the clustered network is displayed, the users can navigate the network in many different ways. Double-clicking on a cluster zooms in on the cluster. Selecting clusters and clicking the *Zoom In *button zooms in on those clusters at once. Selecting nodes and/or clusters and clicking the *Run *button of the *Re-Centering *panel re-centers the network on the selected nodes/clusters (see Implementation). Apart from that, the users can go back and forth between the network views created in the past by using the " < " and " > " buttons. The resultant networks on the canvas can be easily saved as image via Cytoscape's Export menu.

Furthermore, users can configure the settings of NaviClusterCS and switch between domains of interest via the NaviClusterCS menu. General Settings allows for changing the directory that contains domain information (e.g., property information file used in the property-based clustering), a VizMapper file (a file used by Cytoscape to describe visual appearances of nodes, edges, background, etc.), and a graph layout algorithm used when loading networks. Switch Domains allows for selecting domain of interest, changing a property information file, and specifying information about external databases used to create context-specific menus for nodes. For more detail, see the online manual at http://navicluster.cb.k.u-tokyo.ac.jp/cs/.

### Application of NaviClusterCS to the ATTED-II network dataset

We ran NaviClusterCS on ATTED-II, an *Arabidopsis *gene co-expression network dataset with 22,447 nodes and 189,546 edges, which correspond to genes and co-expressions, respectively. The edges were weighted by mutual ranks (MRs) of co-expressions between any two genes [6]. Illustrated in Figure 2 is the abstracted view of the whole ATTED-II network dataset. The number of clusters to be displayed was set at 12. The GO terms assigned to genes were obtained from TAIR [7], and only biological process terms were used for the property-based clustering in this analysis. The numbers shown above the clusters represent the number of nodes within the clusters, and the labels following the numbers are the abbreviated GO terms that get the highest score and, thus, best describe the properties of the clusters. Meta-edges are drawn as blue lines between any two clusters that have at least one edge between at least one member in each of the two clusters, with the numbers next to each edge representing the total numbers of all edges existing between the members of the two clusters. The total numbers are also reflected by the thicknesses of the meta-edges. In addition, a property edge is drawn as a black line between every pair of clusters if they share significant numbers of GO terms.

### Acquiring knowledge about *AT4G18170 *and *AT2G46400 *via *re-centering*

**Figure 3:**
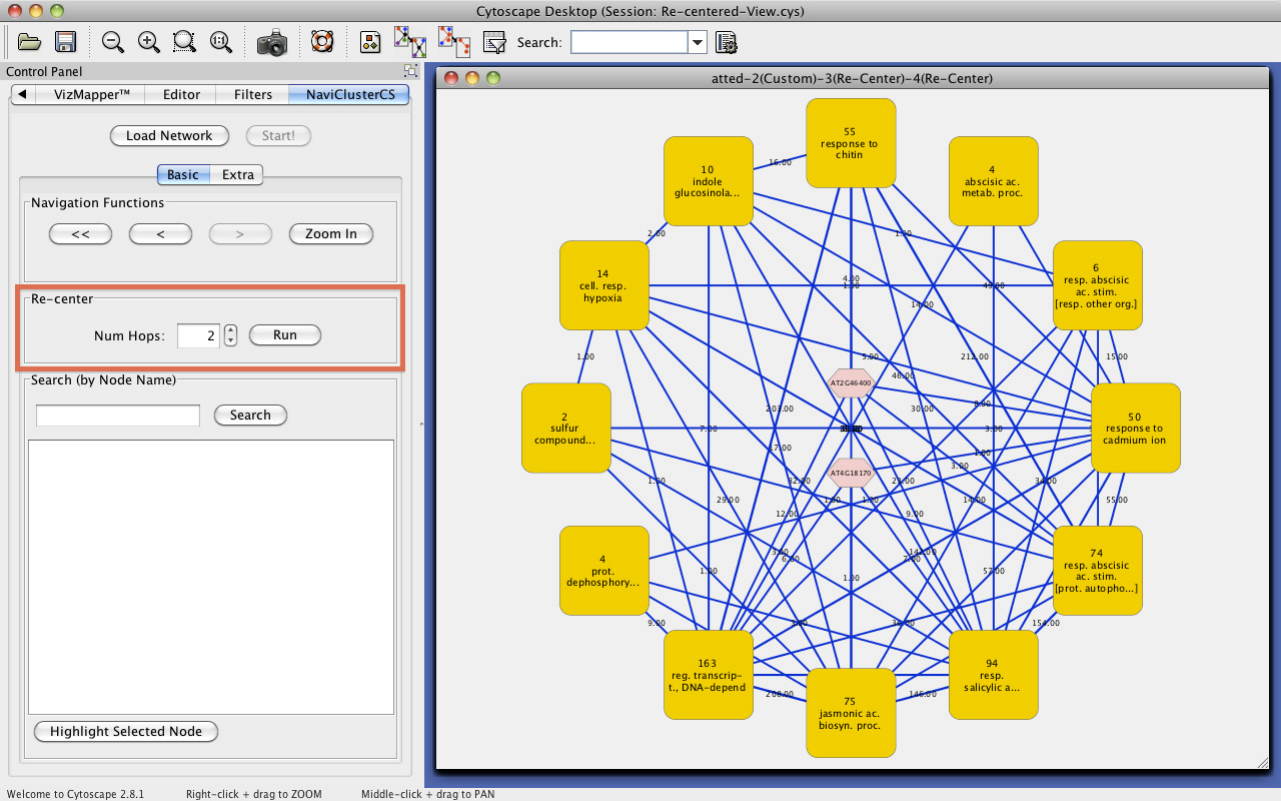
***Re-centered *on *AT4G18170 *and *AT2G46400 *with *Num Hops *set to two**. NaviClusterCS's *re-centering *function allows users to intuitively grasp what types of genes exist around the genes of interest in large networks. In this screenshot, a user selects *AT4G18170 *and *AT2G46400*, sets *Num Hops *to two, and clicks *Run*. Then, NaviClusterCS immediately collects all of the genes that are within two hops from these two genes and clusters them to a visually interpretable level. The user can immediately see clusters related to *response to chitin, response to salicylic acid*, and *response to abscisic acid stimulus*, around the genes of interest, implying that *AT4G18170 *and *AT2G46400 *are involved in defense response mechanisms.

Shown in Figure 3 is a screenshot of NaviClusterCS centered on the genes of interest (in this case, *AT4G18170 *and *AT2G46400*). By invoking its *re-centering *function, a user can grasp what types of genes exist around the genes of interest in this network. In Figure 3, the clusters related to the defense response surround the two genes, suggesting that these genes are also involved in this function. Actually, they have recently been confirmed to be positive regulators of *ICS1 *and *PBS3*, which are key players of systemic acquired resistance (SAR) [18]. It is extremely difficult to infer this kind of knowledge from huge network data if a cluttered visualization is employed; this example clearly illustrates how our visualization approach can provide meaningful and interpretable information for researchers.

### Unraveling hierarchical organization of the network via *zooming*

**Figure 4:**
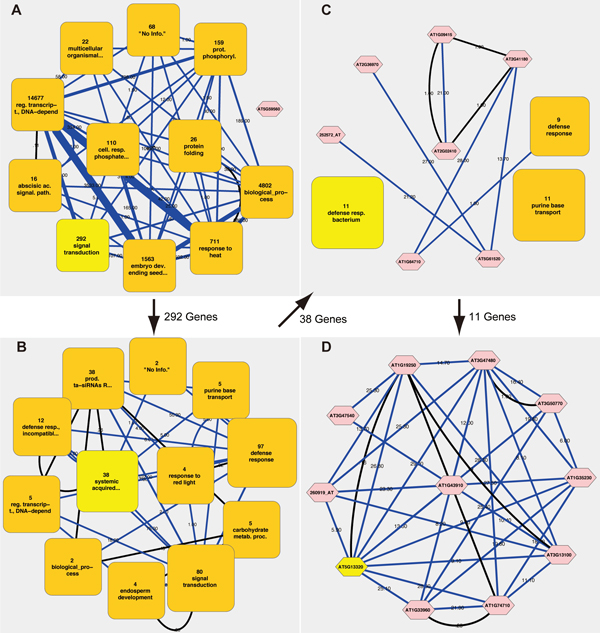
**Multi-scale navigation for *PBS3 *in the ATTED-II network dataset of 22,447 nodes and 189,546 edges**. This figure shows the hierarchical organization of the clusters containing *PBS3*, a gene of interest, which are highlighted in light yellow. (**A**) The most abstracted view. *PBS3 *belongs to the *signal transduction *cluster. (**B**) At this level, *PBS3 *is found in the *systemic acquired resistance *cluster. (**C**) *PBS3 *is included in the *defense response to bacterium *cluster. (**D**) The most specific view. *PBS3 *is clustered together with ten other genes, such as *AIG1, ICS1*, and *FMO1*.

Users can interactively navigate the abstracted network in Figure 2 in a multi-scale manner as illustrated in Figure 4, which shows how the *zooming *function can lead a researcher to the gene of interest (*PBS3 (AT5G13320) *in this case). *PBS3 *encodes a member of an auxin-responsive GH3 family of acyl-adenylate/thioestor-forming enzymes, some of which have been shown to catalyze hormone-amino acid conjugation. In Figure 4, the clusters containing *PBS3 *are highlighted in all of the views; the granularities of detail in each view vary from coarsest to finest.

In Figure 4A, which depicts the entire network, the clusters are labeled with broad biological processes such as *regulation of transcription, DNA-dependent, response to heat*, and *embryo development ending in seed development. PBS3 *is found under the cluster of *signal transduction*, which is highlighted. Indeed, *PBS3 *is involved in a signal transduction cascade of SAR [19]. Zooming in on the *signal transduction *cluster reveals that the *systemic acquired resistance *cluster is highlighted, meaning that *PBS3 *is grouped under this cluster (Figure 4B). This result is in accordance with many sources stating that *PBS3 *affects SAR [18,19]. Zooming in on the *systemic acquired resistance *cluster at this stage indicates that *PBS3 *is a member of the *defense response to bacterium *cluster (Figure 4C). Finally, Figure 4D illustrates the deepest view after zooming in on the *defense response to bacterium *cluster. At this stage, the relationships between *PBS3 *and other genes related to SAR, such as *AT1G74710 (ICS1), AT1G33960 (AIG1)*, and *AT1G19250 (FMO1)*, are depicted [19-21]. This example illustrates that clusters comprising co-expressed genes of similar functions are sensibly created and their roles are indicated informatively and correctly. The amount of information displayed is kept tractable by showing only 12 nodes/clusters per view.

## Conclusions

NaviClusterCS offers interactive and multi-scale network navigation to a wide range of biologists who struggle to acquire knowledge from many types of large networks of functional genomics. As shown in the examples, apart from showing the hierarchical organization of the network under consideration, NaviClusterCS can also assist users in grasping information about nodes (genes) of interest. As a future work, we plan to make NaviClusterCS compatible with Cytoscape 3 and fully take its advantages and new features.

## Availability and requirements

• Project name: NaviClusterCS

• Project home page: http://navicluster.cb.k.u-tokyo.ac.jp/cs/

• Operating system: Platform independent

• Programming language: Java, minimum requirement Java SE 1.6

• Cytoscape version: NaviClusterCS has been tested on version 2.8

• Memory: minimum 1GB

• License: BSD license

• Any restrictions to use by non-academics: none other than those in the BSD license

## List of abbreviations used

OS: Operating System; ATTED-II: *Arabidopsis thaliana trans*-factor and *cis*-element prediction database; MR: Mutual Rank; GO: Gene Ontology; TAIR: The Arabidopsis Information Resource; SAR: Systemic Acquired Resistance; LC: Louvain Cluster; FFT: the Farthest First Traversal *K*-center algorithm.

## Competing interests

The authors declare that they have no competing interests.

## Authors' contributions

TP and WI conceived and designed the experiments. TP implemented software tool and performed the experiments. TP and WI analyzed the data. TP and WI wrote the paper. WI and TT supervised the project.
